# Prognostic value of tumor-infiltrating immune cells and immune checkpoints in elderly head-and-neck squamous cell carcinoma patients undergoing definitive (chemo)radiotherapy

**DOI:** 10.1186/s13014-022-02153-9

**Published:** 2022-11-14

**Authors:** Alexander Rühle, Jovan Todorovic, Simon S. K. Spohn, Eleni Gkika, Christoph Becker, Andreas Knopf, Constantinos Zamboglou, Tanja Sprave, Martin Werner, Anca-Ligia Grosu, Gian Kayser, Nils H. Nicolay

**Affiliations:** 1 Department of Radiation Oncology, Medical Center, Faculty of Medicine, University of Freiburg, Robert-Koch-Str. 3, 79106 Freiburg, Germany; 2 German Cancer Research Center (dkfz), German Cancer Consortium (DKTK) Partner Site Freiburg, Heidelberg, Germany; 3 Institute of Surgical Pathology, Department of Pathology, Medical Center, Faculty of Medicine, University of Freiburg, Freiburg, Germany; 4 Department of Otorhinolaryngology, Medical Center, Faculty of Medicine, University of Freiburg, Freiburg, Germany; 5 Institute of Pathology Naehring Mattern Kayser, Freiburg, Germany; 6 Department of Radiation Oncology, University Hospital Leipzig, Leipzig, Germany

**Keywords:** Head-and-neck cancer, Elderly, Geriatric, Immunosenescence, Immune checkpoint, Tumor-infiltrating lymphocytes, Radiotherapy

## Abstract

**Background and purpose:**

Tumor-infiltrating lymphocytes (TILs) are associated with locoregional control (LRC) in head-and-neck squamous cell carcinoma (HNSCC) patients undergoing (chemo)radiotherapy. As immunosenescence results in reduced immune activity, the role of TILs in elderly HNSCC patients may differ compared to younger patients, providing a rationale to study the prognostic role of TILs and immune checkpoints (ICs) in this population.

**Material and methods:**

Sixty-three HNSCC patients aged ≥ 65 years undergoing definitive (chemo)radiotherapy between 2010 and 2019 with sufficient material from pre-treatment biopsies were included in the analysis. Immunohistochemical stainings of CD3, CD4, CD8, PD-L1, TIM3, LAG3, TIGIT and CD96, and of osteopontin as an immunosenescence-associated protein were performed. Overall survival (OS) and progression-free survival (PFS) were determined using the Kaplan–Meier method, and Fine-Gray's models were used for locoregional failure (LRF) analyses.

**Results:**

While there was no correlation between patient age and IC expression, osteopontin levels correlated with increasing age (*r* = 0.322, *p* < 0.05). Two-year OS, PFS, and LRC were 44%, 34%, and 71%, respectively. Increased LAG3 expression, both intraepithelial (SHR = 0.33, *p* < 0.05) and stromal (SHR = 0.38, *p* < 0.05), and elevated stromal TIM3 expression (SHR = 0.32, *p* < 0.05) corresponded with reduced LRFs. Absent tumoral PD-L1 expression (TPS = 0%) was associated with more LRFs (SHR = 0.28, *p* < 0.05). There was a trend towards improved LRF rates in elderly patients with increased intraepithelial CD3 + (SHR = 0.52, *p* = 0.07) and CD8 + (SHR = 0.52, *p* = 0.09) TIL levels.

**Conclusion:**

LAG3, TIM3 and TPS are promising biomarkers in elderly HNSCC patients receiving (chemo)radiotherapy. Considering the frequency of non-cancer related deaths in this population, the prognostic value of these biomarkers primarily relates to LRC.

**Supplementary Information:**

The online version contains supplementary material available at 10.1186/s13014-022-02153-9.

## Introduction

With about 450,000 deaths per year, head-and-neck squamous cell carcinoma (HNSCC) constitutes a relevant global health issue [1, 2]. There is a continuous increase of elderly HNSCC patients for whom treatment concepts of younger patients cannot simply be extrapolated, as elderly patients exhibit significant clinical and tumor-related differences and have been underrepresented or excluded from most treatment-defining clinical trials [3–7]. Radiotherapy, either alone or with concomitant chemotherapy, constitutes a key treatment for HNSCC [5, 6, 8, 9]. Identification of prognostic biomarkers is crucial, as they could aid treatment decision-making, facilitate trial conception and contribute to developing personalized radiotherapy approaches for elderly HNSCC patients [10]. Genes and proteins related to HPV status, tumor hypoxia, the (anti-)tumoral immune system and cancer stem cells are examples of biomarkers that have been studied for HNSCC patients undergoing (chemo)radiotherapy [11–15]. Previous analyses and systematic meta-analyses have shown that higher levels of tumor-infiltrating lymphocytes (TILs), especially CD3 + and CD8 + TILs, are associated with improved survival in HNSCC patients receiving (chemo)radiotherapy [16–23]. In contrast, the prognostic role of immune checkpoints (ICs) for HNSCC patients undergoing radiotherapy is less clear [24–31]. Nevertheless, the tumoral PD-L1 status may become more relevant in the future considering clinical approaches combining (chemo)radiotherapy with IC inhibitors [32].

Immunosenescence, the process of immune dysfunction occurring with increasing age, includes reductions of T cells’ anti-tumor abilities, upregulated expression of ICs in immune cells, and elevated secretion of molecules belonging to the senescence-associated secretory phenotype such as osteopontin [33]. Therefore, immunosenescence may abrogate the prognostic value of TILs in elderly HNSCC patients and could in turn reveal new biomarkers, e.g., ICs. However, little is known about the relevance of immunosenescence in HNSCC patients [34–36].

Therefore, we aimed to analyze the prognostic role of TILs and ICs for elderly HNSCC patients undergoing definitive (chemo)radiotherapy and the alterations in TIL levels and IC expression with increasing patient age. CD3 was chosen to analyze the levels of tumor-infiltrating T lymphocytes, while CD4 and CD8 stainings reflect the levels of tumor-infiltrating T helper cells and cytotoxic T lymphocytes, respectively. The expression of PD-L1, the ligand of the PD-1 receptor, was studied, as the PD-1/PD-L1 axis is involved in cancer immune escape which can be targeted by IC inhibitors such as pembrolizumab and nivolumab, that are clinically used in recurrent or metastatic HNSCC patients [37, 38]. TIM3, LAG3 and CD96 are other ICs that are intensively studied as potential targets in preclinical studies and clinical trials [39–43]. While plasma levels of osteopontin, a bone sialoprotein, have shown to exhibit predictive value regarding tumor hypoxia and the potential benefit of the hypoxia modifier nimorazole [44, 45], osteopontin was also found to be abundantly secreted by immunosenescent T cells [46, 47].

## Materials and methods

### Patients and treatment

Elderly patients (≥ 65 years) receiving definitive (chemo)radiotherapy between 2010 and 2019 at the Department of Radiation Oncology, University of Freiburg – Medical Center were analyzed regarding the prognostic value of TILs, ICs and osteopontin. The presence of distant metastases at the time of (chemo)radiotherapy was considered as an exclusion criterion. Demographic and treatment characteristics were obtained from the electronic patient records. The age-adjusted Charlson Comorbidity Index (CCI) was calculated as described in the literature [48], whereby HNSCC itself was not included in the CCI. The 7th edition of the TNM staging system was used as staging system for all analyzed patients.

Treatment decisions were based on multidisciplinary tumor board recommendations. Patients were treated with 66–70 Gy EQD2 (*α*/*ß* = 10) to the macroscopic tumor and 50 to 60 Gy EQD2 to the low-risk and intermediate target volumes including the elective cervical lymphatic drainage depending on their tumor stage. Elective lymph node stations were delineated based on the international consensus guidelines, and treatment was performed in five weekly fractions [49, 50]. In case of no contraindications against cisplatin, patients with locally advanced HNSCCs received 2–3 cycles of high-dose cisplatin (100 mg/m^2^) or alternatively 6–7 weekly cisplatin administrations (40 mg/m^2^). Patients underwent follow-up consultations including cross-sectional imaging of the head-and-neck region three-monthly for the first 2 years. After 2 years, the follow-up intervals were extended to 6–12 months.

The present analysis was approved by the institutional review board of the University of Freiburg (reference no. 551/18).

### Immunohistochemical stainings

Paraffin embedding, sectioning, mounting, deparaffinization, rehydration and heat-induced antigen retrieval was performed as reported previously [20, 24]. Additional file 1: STable 1 includes details of primary antibodies. The HPV status was assessed using the HPV-Type 3.5 LCD-Array (Chipron, Berlin, Germany). The expression of osteopontin was examined semi-quantitatively using the H-score. TIL levels were trichotomized into three groups: (a) ≤ 20 TILs/high power field (HPF), (b) 21–100 TILs/HPF and (c) > 100 TILs/HPF. TIL and IC expression was calculated both for the intraepithelial and stromal compartment. Following international guidelines, the tumor proportion score (TPS) was defined as the ratio of PD-L1-positive tumor cells divided by the total number of viable tumor cells multiplied by 100. The combined positive score (CPS) was calculated by dividing the sum of PD-L1-positive tumor cells and immune cells by the total number of viable tumor cells multiplied by 100 (if exceeding 100, the value was set to 100). All stainings were analyzed by two independent pathologists, and both pathologists were blinded to the oncological outcomes of the analyzed patients.

### Statistics

The association between PD-L1 expression or osteopontin levels with age was analyzed using Pearson’s correlations. Regarding the association between age and TIL levels, groups were compared with Mann–Whitney-U tests (for 2 groups) or Kruskal–Wallis tests (for 3 groups).

Overall survival (OS) was computed from the start of (chemo)radiotherapy until death, and progression-free survival (PFS) was calculated from the start of (chemo)radiotherapy until death, local/locoregional progression or development of distant metastases. Locoregional control (LRC) was defined as absence of local or locoregional progression. Patients were censored at the last follow-up date, and missing survival data were obtained from the Comprehensive Cancer Center Freiburg. Survival analyses were performed based on the Kaplan–Meier method, and survival curves were compared using log-rank tests. Cox proportional hazards regression analyses were performed for OS and PFS, and hazard ratios (HR) with the corresponding 95% confidence intervals (95% CI) were shown. Considering death as a competing risk for locoregional failure (LRF), Fine and Gray's proportional subhazards models were used for LRF analyses, and subhazard ratios (SHRs) were calculated. A *p*-value of < 0.05 was considered statistically significant for all analyses. SPSS Statistics version 25 (IBM, Armonk, NY, USA), Stata version 16 (StataCorp LP, College Station, TX, USA) and GraphPad version 8.2.1 (GraphPad Software, San Diego, CA, USA) were used for statistical analyses and visualization.

## Results

**Fig. 1:**
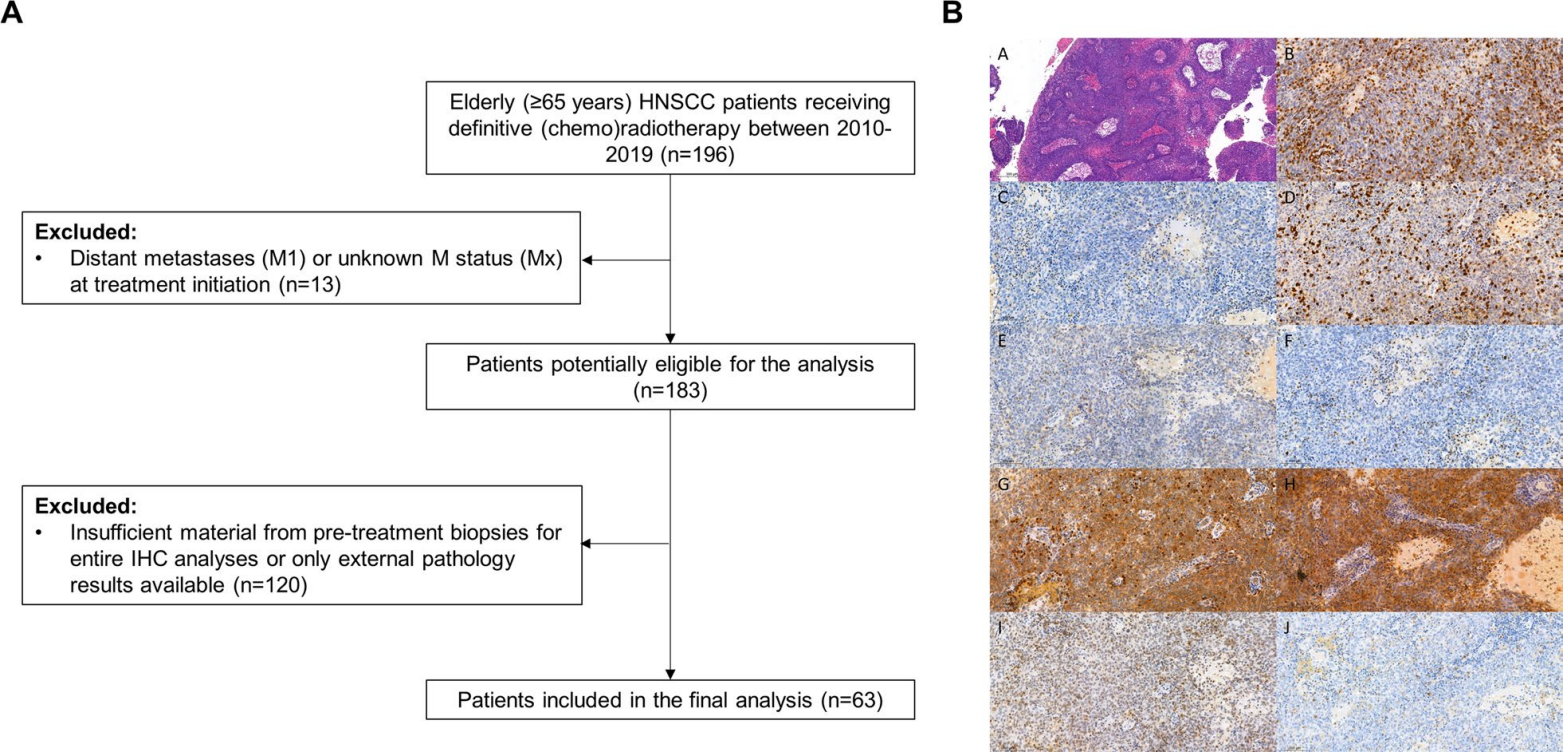
CONSORT diagram and representative immunohistochemistry stainings showing the workflow of the study. **A** CONSORT diagram showing the inclusion and exclusion process of the analyzed cohort. **B** Representative hematoxylin and eosin staining **A** as well as IHC stainings of the TIL markers CD3 **B**, CD4 **C** and CD8 **D**, and the ICs/immunosenescence-associated proteins CD96 **E**, LAG3 **F**, osteopontin **G**, PD-L1 **H**, TIGIT **I** and TIM3 **J**. Hematoxylin and eosin staining: 10 × magnification, IHC stainings: 20 × magnification

A total of 63 patients with a median age of 73 years (range 65–96 years) met the inclusion criteria and were included in this analysis (Fig. 1, Table 1). Median CCI amounted to 4 points (range 2–9), and the most common ECOG performance status was 0 (*n* = 31, 49.2%). The majority of patients was male (*n* = 48, 76.2%) and classified as smokers (*n* = 42, 66.7%). Most common tumor localizations were oropharynx (*n* = 26, 41.3%), larynx (*n* = 13, 20.6%) and hypopharynx (*n* = 12, 19.0%). The vast majority of HNSCCs presented with locally advanced cancers (UICC III-IV: *n* = 55, 87.3%). Thirteen patients (20.6%) suffered from HPV-positive oropharyngeal cancers. Concomitant chemotherapy was administered in 44 patients (69.8%).

**Table 1:** Demographic and treatment characteristics of the analyzed cohort

	Median (range)		
Age		73 (65–96)	
Age-adjusted CCI		4 (2–9)	
		n	%
Gender	Male	48	76.2
	Female	15	23.8
Smoking	Non-smoker	17	27.0
	Smoker	42	66.7
	Missing	4	6.3
ECOG	ECOG 0	31	49.2
	ECOG 1	27	42.9
	ECOG 2	5	7.9
Localization	Nasopharynx	3	4.8
	Oropharynx	26	41.3
	Hypopharynx	12	19.0
	Oral cavity	7	11.1
	Larynx	13	20.6
	Multi-level	1	1.6
	Parotid gland	1	1.6
UICC	I	6	9.5
	II	2	3.2
	III	5	7.9
	IV	50	79.4
cT	cT1	7	11.1
	cT2	10	15.9
	cT3	16	25.4
	cT4	30	47.6
cN	cN0	16	25.4
	cN1	3	4.8
	cN2a	1	1.6
	cN2b	25	39.7
	cN2c	15	23.8
	cN3	3	4.8
cM	cM0	63	100.0
	cM1	0	0.0
Grading	G1	2	3.2
	G2	43	68.3
	G3	18	28.6
HPV	HPV-negative	25	39.7
	HPV-positive	13	20.6
	Missing	25	39.7
		Median (range)	
Dose (EQD2)		69.4 Gy (40.0–70.0 Gy)	
		*n*	%
Radiotherapy completed	Not completed	11	17.5
	Completed	52	82.5
Chemotherapy	No chemotherapy	19	30.2
	Chemotherapy	44	69.8

A positive smoking status was considered in case of ≥ 10 pack years. Staging was performed using the 7th edition of the TNM classification

**Fig. 2:**
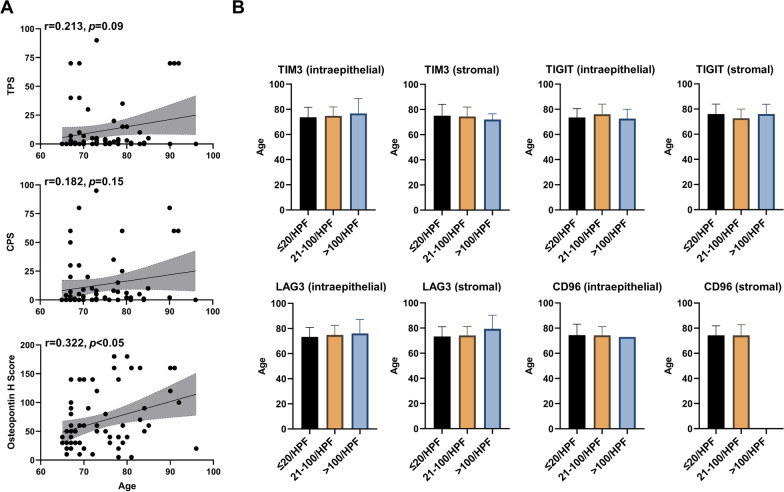
Osteopontin weakly correlates with increasing age. **A** XY-diagram showing the association between age and TPS, CPS and osteopontin. Pearson’s r values with the according *p*-values and regression lines with the corresponding 95% CI are displayed. **B** Association between patient age and expression of the ICs TIM3, LAG3, TIGIT and CD96, measured both intraepithelial and stromal. Mean values with the according standard deviations are shown. Groups were compared with Mann–Whitney-*U* tests (for 2 groups) or Kruskal–Wallis tests (for 3 groups)

Both IC and osteopontin expression have been reported to be associated with immunosenescence [51, 52]. Twenty-six patients (41.3%) exhibited a TPS of 0% and 53 patients (84.1%) a TPS < 25% (Table 2) which was a relevant threshold in a previous HNSCC trial investigating the value of concomitant IC-inhibitor treatment [32]. One third of the population (*n* = 21, 33.3%) had a CPS of 0, and 49 patients (77.8%) a CPS below 20, another clinically relevant threshold [37]. There was no significant correlation between patient age and the PD-L1 expression on tumor and immune cells (i.e., CPS: *r* = 0.182, *p* = 0.15) (Fig. 2). While the TPS was also not associated with patient age (*r* = 0.213, *p* = 0.09), osteopontin levels significantly correlated with increasing age (*r* = 0.322, *p* < 0.05). None of the other analyzed ICs, both intraepithelial and stromal, were related to age.

**Table 2:** CPS and TPS within elderly HNSCC patients receiving definitive (chemo)radiotherapy

TPS (%)	*n*	%
0	26	41.3
1–24	27	42.9
25–49	4	6.3
≥ 50	6	9.5
CPS		
0	21	33.3
1–19	28	44.4
20–49	6	9.5
≥ 50	8	12.7

The relationship between patient age and TIL levels was analyzed by comparing the mean ages in dependence of TIL levels using Mann–Whitney-*U* or Kruskal–Wallis tests (Table 3). Neither CD3 + (intraepithelial: *p* = 0.42, stromal: *p* = 0.31) nor CD4 + TIL levels (intraepithelial: *p* = 0.21, stromal: *p* = 0.50) differed depending on patient age. However, the mean age of patients with high stromal CD8 + TIL levels was significantly higher than in patients with low levels (76 vs. 72 years, *p* < 0.05), whereas there was no age association for intraepithelial CD8 + TIL levels (*p* = 0.41).

**Table 3:** TIL levels depending on patient age

Variable	HR	Mean age (SD)	*p*
CD3 + TILs (intraepithelial)	≤ 20/HPF (*n* = 5)	79 (11)	
	21–100/HPF (*n* = 34)	74 (7)	
	> 100/HPF (*n* = 24)	76 (8)	0.42^a^
CD3 + TILs (stromal)	21–100/HPF (10)	76 (8)	
	> 100/HPF (53)	74 (7)	0.31^b^
CD4 + TILs (intraepithelial)	≤ 20/HPF (*n* = 42)	74 (7)	
	21–100/HPF (20)	75 (8)	
	> 100/HPF (*n* = 1)	92	0.21^a^
CD4 + TILs (stromal)	≤ 20/HPF (*n* = 19)	73 (8)	
	21–100/HPF (*n* = 30)	75 (8)	
	> 100/HPF (*n* = 14)	75 (7)	0.50^a^
CD8 + TILs (intraepithelial)	≤ 20/HPF (*n* = 15)	73 (8)	
	21–100/HPF (*n* = 33)	74 (7)	
	> 100/HPF (*n* = 15)	76 (8)	0.41^a^
CD8 + TILs (stromal)	21–100/HPF (*n* = 30)	72 (7)	
	> 100/HPF (*n* = 33)	76 (8)	< 0.05^b^

^a^ Kruskal–Wallis test

^b^ Mann–Whitney-*U* test

Mean age depending on the levels of TILs intraepithelial and stromal. Mean age including standard deviation is shown. Groups were compared using Mann–Whitney-*U* tests (for 2 groups) or Kruskal–Wallis tests (for 3 groups)

Median follow-up was 40 months, as calculated using the reverse Kaplan–Meier method. At the time of analysis, 41 deaths (65.1%), 13 locoregional failures (20.6%) and 7 distant metastases (11.1%) had occurred. Two-year estimates for OS, PFS, and LRC were 44.2%, 34.4%, and 70.9%, respectively. Median OS and PFS amounted to 21 months and 10 months, respectively, while median LRC was not reached.

**Fig. 3:**
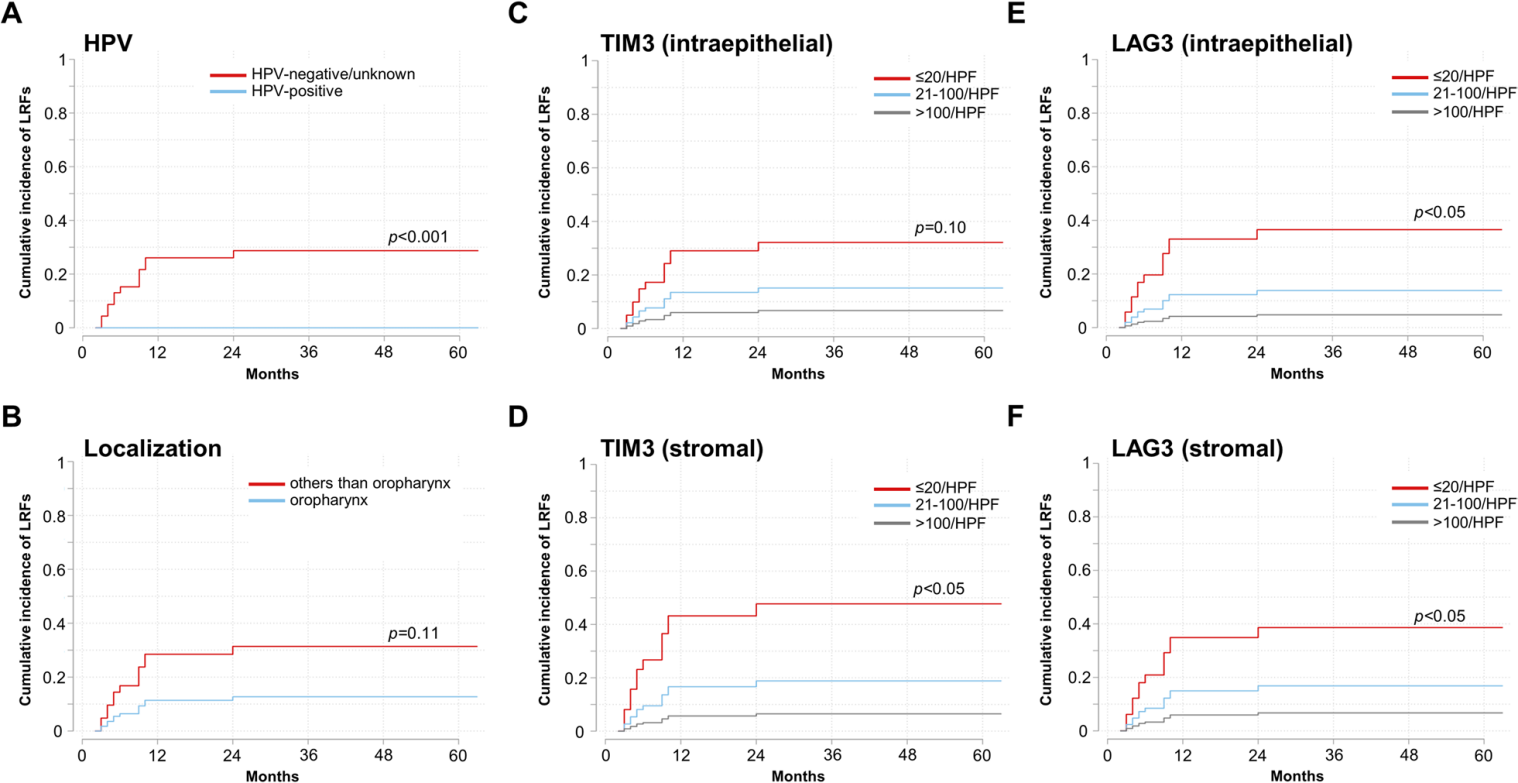
The ICs TIM3 and LAG3 are associated with LRC. Cumulative incidence of LRFs regarding HPV status **A**, localization **B**, intraepithelial **C** and stromal **D** TIM3 expression as well as intraepithelial **E** and stromal **F** LAG3 expression based on Fine and Gray’s proportional subhazards models are shown

Due to the scope of our analysis, only tumor-related variables were analyzed regarding their impact on oncological outcomes. Cox analyses revealed oropharyngeal tumor localization as a prognosticator for superior OS (HR = 0.36, 95% CI 0.18–0.72, *p* < 0.01) (Table 4). Patients with HPV-positive HNSCCs exhibited a trend towards superior PFS (HR = 0.43, 95% CI 0.17–1.09, *p* = 0.08). None of the analyzed TIL and IC markers were associated with improved OS or PFS. Considering the high prevalence of competing risks regarding LRF (23 competing events in the LRF analysis), Fine and Gray's models were used to attribute for this competition. None of the HPV-positive oropharyngeal cancers recurred locoregionally, translating into a significantly reduced LRF risk (SHR < 0.001, *p* < 0.001; Fig. 3). Both increased TIM3 (intraepithelial: SHR = 0.42, 95% CI 0.15–1.18, *p* = 0.10, stromal: SHR = 0.32, 95% CI 0.14–0.76, *p* < 0.05) and LAG3 (intraepithelial: SHR = 0.33, 95% CI 0.12–0.93, *p* < 0.05, stromal: SHR = 0.38, 95% CI 0.15–0.95, *p* < 0.05) expression corresponded to a lower risk for LRFs.

**Table 4:** Prognostic role of TILs and ICs in elderly HNSCC patients undergoing (chemo)radiotherapy

	OS			PFS			LRF		
Variable	HR	CI 95%	*p*	HR	CI 95%	*p*	SHR	CI 95%	*p*
HPV (reference: HPV-negative)	0.48	0.19–1.23	0.13	0.43	0.17–1.09	0.08	< 0.001	< 0.001- < 0.001	** < 0.001**
Localization (reference: non-oropharynx)	0.36	0.18–0.72	** < 0.01**	0.37	0.19–0.73	** < 0.01**	0.36	0.10–1.27	0.11
CD3 + TILs (intraepithelial)	0.99	0.58–1.70	0.98	0.88	0.53–1.46	0.62	0.52	0.26–1.06	0.07
CD3 + TILs (stromal)	1.06	0.45–2.52	0.90	0.69	0.32–1.50	0.35	0.41	0.14–1.23	0.11
CD4 + TILs (intraepithelial)	1.20	0.66–2.16	0.55	0.99	0.55–1.76	0.96	0.57	0.17–1.92	0.37
CD4 + TILs (stromal)	0.89	0.59–1.36	0.60	0.80	0.53–1.22	0.31	0.53	0.24–1.17	0.12
CD8 + TILs (intraepithelial)	1.12	0.71–1.75	0.63	0.93	0.60–1.44	0.73	0.52	0.25–1.10	0.09
CD8 + TILs (stromal)	1.53	0.83–2.85	0.18	1.27	0.69–2.31	0.45	0.67	0.22–1.98	0.47
TPS (continuous)	1.39	0.34–5.73	0.65	1.01	0.25–4.11	0.99	0.96	0.90–1.03	0.22
TPS (reference: TPS = 0)	1.03	0.55–1.93	0.94	0.81	0.44–1.50	0.51	0.28	0.09–0.93	** < 0.05**
CPS (continuous)	1.00	0.99–1.02	0.87	1.00	0.98–1.01	0.79	0.94	0.88–1.01	0.08
CPS (reference: CPS = 0)	0.97	0.51–1.83	0.92	0.85	0.46–1.58	0.61	0.34	0.11–1.01	0.05
TIM3 (intraepithelial)	1.06	0.64–1.75	0.84	0.95	0.57–1.58	0.84	0.42	0.15–1.18	0.10
TIM3 (stromal)	0.81	0.46–1.43	0.47	0.72	0.41–1.27	0.26	0.32	0.14–0.76	** < 0.05**
LAG3 (intraepithelial)	1.16	0.70–1.90	0.57	0.96	0.58–1.58	0.87	0.33	0.12–0.93	** < 0.05**
LAG3 (stromal)	0.71	0.41–1.24	0.23	0.68	0.40–1.16	0.15	0.38	0.15–0.95	** < 0.05**
TIGIT (intraepithelial)	1.12	0.74–1.70	0.60	1.05	0.68–1.61	0.84	0.62	0.27–1.39	0.25
TIGIT (stromal)	1.31	0.77–2.24	0.32	1.10	0.66–1.83	0.72	0.54	0.25–1.16	0.11
CD96 (intraepithelial)	1.96	0.81–4.74	0.14	1.49	0.63–3.56	0.37	0.57	0.07–4.49	0.60
CD96 (stromal)	0.98	0.54–1.80	0.95	0.79	0.44–1.42	0.43	1.00	0.40–2.52	0.99
Osteopontin^a^ (continuous)	1.00	0.99–1.00	0.48	1.00	0.99–1.00	0.32	1.00	0.98–1.01	0.53

Bold indicate *p*-values < 0.05

Cox proportional hazard regression analyses were performed regarding OS and PFS, while the Fine and Gray's proportional subhazards model was used for LRF analyses considering the presence of competing risks

^a^ Osteopontin H score

TPS values > 0 were associated with a significantly lower risk for LRFs (SHR = 0.28, 95% CI 0.09–0.93, *p* < 0.05). Similarly, elderly HNSCC patients with a CPS > 0 tended to experience a reduced risk for LRFs (SHR = 0.34, 95% CI 0.11–1.01, *p* = 0.05).

**Fig. 4:**
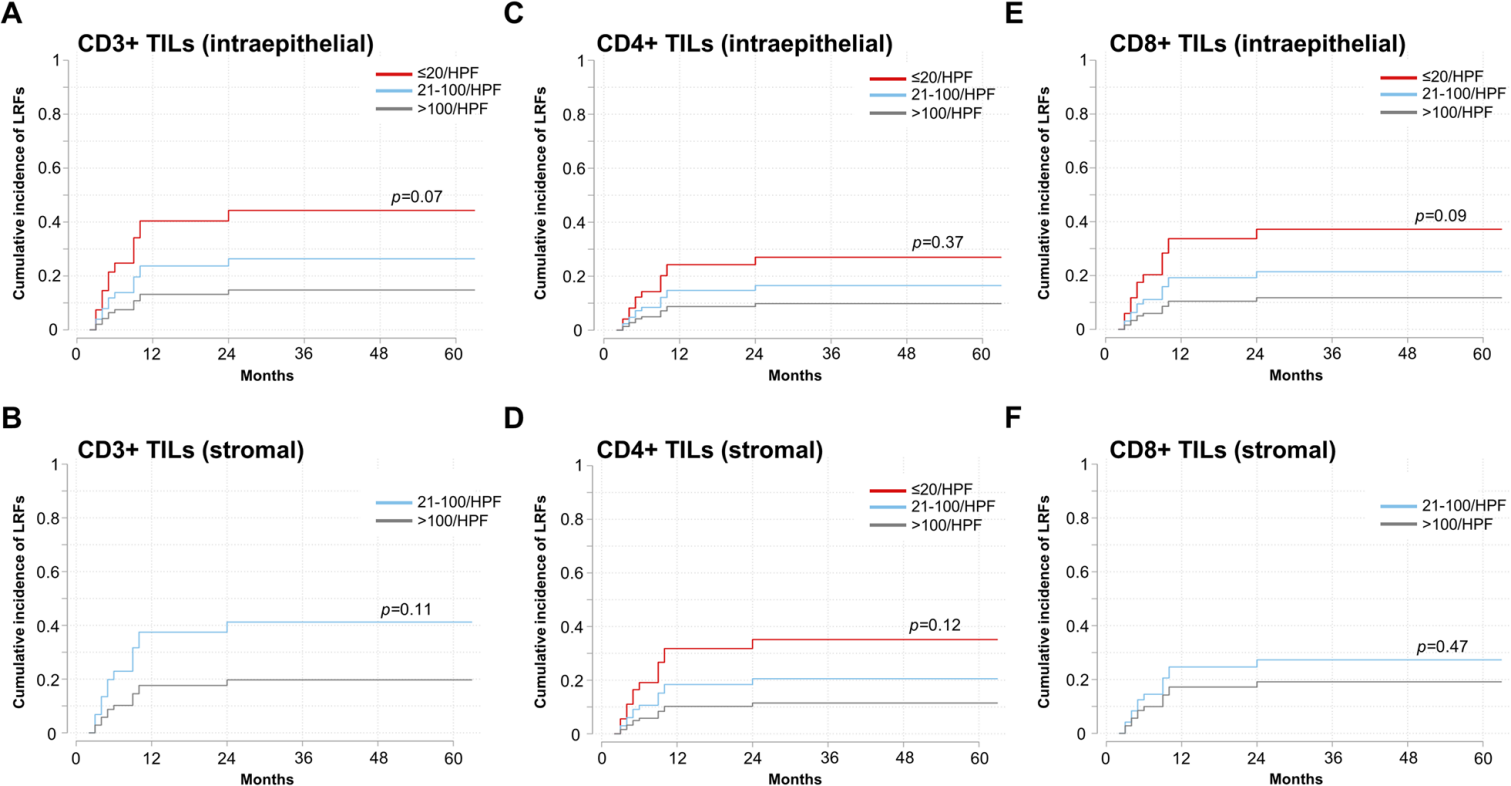
Increased intraepithelial CD3 + and CD8 + TIL levels result in a trend towards superior LRC. Cumulative incidence of LRFs regarding CD3 + (**A** and **B**), CD4 + (**C** and **D**) and CD8 + TIL levels (**E** and **F**) based on Fine and Gray’s proportional subhazards models are shown

HNSCCs with high CD3 + TIL levels exhibited reduced LRF rates, although this correlation did not reach statistical significance (intraepithelial: SHR = 0.52, 95% CI 0.26–1.06, *p* = 0.07, stromal: SHR = 0.41, 95% CI 0.14–1.23, *p* = 0.11) (Fig. 4). While higher CD4 + TIL levels did not translate into improved LRC in our cohort (intraepithelial: SHR = 0.57, 95% CI 0.17–1.92, *p* = 0.37, stromal: SHR = 0.53, 95% CI 0.24–1.17, *p* = 0.12), there was a trend towards improved LRC for patients with higher intraepithelial CD8 + TIL numbers (SHR = 0.52, 95% CI 0.25–1.10, *p* = 0.09). In contrast, stromal CD8 + TILs were not associated with the cumulative incidence of LRFs (SHR = 0.67, 95% CI 0.22–1.98, *p* = 0.47).

## Discussion

To the best of our knowledge, this is the first study regarding the prognostic value of TILs and IC expression exclusively in elderly HNSCC patients undergoing definitive (chemo)radiotherapy. The ICs LAG3, TIM3 and PD-L1 were identified as prognosticators for LRC. In consideration of the potential benefit of LRC rates in patients with increased intraepithelial CD3 + and CD8 + TIL levels, there is a need for further studies with larger patient numbers. Importantly, the identified biomarkers only provided prognostic value regarding LRC but not survival, most likely due to the frequent non-cancer related deaths in this vulnerable population.

The number of intraepithelial CD8 + TILs has been shown to positively correlate with oncological outcomes for several tumor entities [53–55]. In HNSCC, previous studies and systematic meta-analyses have also shown a positive prognostic role of CD3 + and CD8 + TILs [16–23]. In our study, CD3 and CD8 expression correlated with locoregional control in elderly patients with a SHR of 0.52, although the associations did not reach statistical significance, most likely due to the limited cohort size. In comparison, the HR of the recent meta-analysis by Borsetto et al. amounted to 0.64 for CD8 + TILs regarding OS [23]. Our findings provide a rationale to carry out further studies to reveal the exact prognostic role of these TIL markers in the elderly HNSCC population.

Jeske and colleagues conducted a comprehensive study concerning age-related changes in T cell populations, both within the blood and in the tumor, among healthy and HNSCC individuals [34]. The frequency of tumor-infiltrating T_reg_ cells and the expression of the ectonucleotidase CD73 producing the immunosuppressive molecule adenosine was lower, while PD1 expression on T cells was positively correlated with increasing age both in healthy volunteers and in HNSCC patients. The authors concluded that there were signs of immunosenescence in elderly HNSCC patients. Importantly, the frequency of CD8 + TILs was found stable in their elderly HNSCC patient cohort. While TIL levels were assessed by flow cytometry in that study, we conducted IHC analyses that provided separate information for the intraepithelial and stromal compartment.

While we could not observe a significant correlation between the ICs PD-L1, TIM3, LAG3, TIGIT and CD96 with increasing age, osteopontin levels were weakly but significantly associated with higher age in our study. Osteopontin is involved in several pathways related to proliferation, angiogenesis and immunosuppression in cancers including HNSCC [56]. Furthermore, both intratumoral and plasma osteopontin have been linked to tumor hypoxia, and secretion of osteopontin is increased in senescence-associated T cells, therefore considered as immunosenescence marker [44, 45, 47, 51, 57]. To the best of our knowledge, this is the first report about an association between tissue osteopontin levels and patient age among elderly HNSCC patients. In the DAHANCA 5 trial cohort, median age was comparable between the osteopontin_low_ and osteopontin_high_ group [45]. Further studies however are required to examine the exact association between osteopontin and age in HNSCC patients.

In our study, the ICs LAG3, TIM3 and tumoral PD-L1 were associated with LRC. LAG3 is mostly expressed on activated T cells and inhibits T cell activation by interacting with several ligands [58, 59]. Studies in which LAG3 is targeted, mostly in combination with other IC inhibitors, are currently conducted for several tumor types such as melanoma, multiple myeloma (NCT04150965), colon cancer (NCT03978611) or lung cancer (NCT04623775) [42]. The prognostic role of LAG3 is controversial and seems to be dependent on the tumor entity, treatment type, levels of TILs, site of expression (intraepithelial vs. invasive tumor margin vs. tumor stroma) and co-expression of other ICs. For instance, LAG3 was reported to be associated with poor OS in colorectal cancer and non-small cell lung cancer, whereas it was a favorable prognostic marker in gastric cancer [60–62]. There are few studied that investigated the role of LAG3 in HNSCC [41, 63]. In the study of Deng and colleagues, increased LAG3 expression went along with diminished survival; however, up-front surgery was the predominant treatment modality [63]. The analysis of Botticelli et al. examined soluble LAG3 within HNSCC patients’ blood and revealed a negative prognostic role of this parameter for PFS and OS [41]. Due to the differences in treatment type and analysis method, comparisons to our study are complicated.

TIM3 that positively correlated with superior LRC in our analysis is expressed on several immune cells such as T cells, T_regs_, dendritic cells and macrophages [58]. It can decrease activated T cells’ proliferation and effector cytokine secretion, thereby serving as regulator of CD8 + T‐cell exhaustion [58]. In pancreatic cancer, TIM3 expression was positively associated with survival, while in early breast cancer, higher TIM3 levels went along with increased cancer-specific survival [64, 65]. In a study of Yang et al., in which the TIM3 expression was analyzed in 80 HNSCC patients all treated by surgery, higher number of TIM3 + TILs correlated with reduced OS [66]. Another study however could not find an association between TIM3 expression, assessed by IHC, and survival in HNSCC patients [39]. Concerning the heterogeneous reports, further studies including systematic (meta-)analyses are required to elaborate the prognostic value of TIM3 in HNSCC.

There are conflicting data regarding the prognostic value of tumoral PD-L1 expression in HNSCC [24–31]. Within a multicenter study of the German Cancer Consortium Radiation Oncology Group, Balermpas et al. showed a positive relationship between elevated PD-L1 expression and survival in locally advanced HNSCC patients treated by postoperative chemoradiotherapy [25]. In another study, only high PD-L1 expression on immune cells but not on tumor cells was found to correspond with superior survival in HNSCC patients receiving surgery [29]. A recent meta-analysis showed favorable outcomes regarding increased tumoral PD-L1 expression in oropharyngeal cancer patients, both after surgery and primary (chemo)radiotherapy [27]. In line with the result of this meta-analysis, our study (in which more than 40% of patients suffered from oropharyngeal cancer) showed favorable LRC rates in patients with positive TPS.

In the future, IC inhibition plus radiotherapy may become an attractive alternative to definitive chemoradiotherapy in elderly HNSCC patients concerning the reducing benefit of concomitant chemotherapy in the elderly and the considerably burden of chemotherapy-related toxicities [67, 68]. In this context, our study also provides information concerning the TPS and CPS in elderly HNSCC patients: 41% of patients exhibited a negative TPS and 84% a TPS < 25%, showing that only a minority of patients may benefit from a combination treatment as demonstrated by the subgroup analysis of the JAVELIN Head and Neck 100 trial [32]. Similarly, only 22% exhibited a CPS ≥ 20, a threshold that indicated benefit of single-agent pembrolizumab compared to multi-agent chemotherapy in recurrent or metastatic HNSCC patients [37]. Studies regarding the association between PD-L1 expression and age are rare: One retrospective study demonstrated that CPS positivity was higher in elderly gastric cancer patients than in their younger counterparts [69]. Consistent with this, another study also showed a positive correlation between PD-L1 expression and age within a large dataset of 968 gastric cancer patients [70]. Smaller studies of HNSCC patients could not reveal a relationship between age and PD-L1 expression, thereby supporting our data [71–73].

Despite presenting a comprehensive analysis of several TIL and IC markers in elderly HNSCC patients, our study exhibits some limitations. As patients did not undergo surgery, only small tissue specimens from biopsies were available; these samples may not represent the complete tumor due to the well-known intratumor heterogeneity. Furthermore, differences related to the IHC assays and the applied antibodies were found to exist for PD-L1 analyses, therefore complicating comparisons to other studies [74]. As the scope of our analyses was to investigate IC and TIL markers within a broad range of elderly HNSCC patients (65–96 years), our analysis does not provide direct comparative analyses between younger and older HNSCC patients.

In the future, studies with larger patient numbers are required to compare TIL levels and IC expression between elderly and younger HNSCC patients. Comparative analyses of peritherapeutic alterations in circulating immune cells during (chemo)radiotherapy may complement baseline IHC stainings in this context [75]. Post-hoc analyses of studies in which IC inhibitors are administered prior to surgery (e.g., KEYNOTE-689 [76]) could also reveal potential differences between elderly and younger HNSCC patients (e.g., regarding tumor regression or changes in TIL levels before and after IC inhibitor administration). Given the increasing number of elderly HNSCC patients on the one side and the lack of current data about the impact of immunosenescence on treatment outcomes in elderly HNSCC patients on the other side, huge efforts are required in the future to increase the scientific knowledge on this issue.

In conclusion, we could demonstrate a favorable prognostic role of LAG3, TIM3 and PD-L1 in elderly HNSCC patients treated by definitive (chemo)radiotherapy in terms of LRC. Our results regarding the absent prognostic role of the analyzed IC and TIL markers for PFS and OS, likely related to the frequency of non-cancer related deaths in this population, highlight the importance of incorporating patient-related parameters such as performance status and comorbidities to accurately assess elderly HNSCC patients’ prognosis beyond LRC [77].

## Supplementary Information

**Additional file 1: Table S1: **Details about the antibodies used in the study.

## Data Availability

The datasets supporting the conclusions of this article are available from the corresponding author on reasonable request.
