# Supplementary material for: Prognostic value of tumor-infiltrating immune cells and immune checkpoints in elderly head-and-neck squamous cell carcinoma patients undergoing definitive (chemo)radiotherapy
Source: Radiat Oncol. 2022 Nov 14;17:181. doi: 10.1186/s13014-022-02153-9 (PMC9661751; doi:10.1186/s13014-022-02153-9)
Supplement: Supplementary file 1 — Additional file 1: Table S1: Details about the antibodies used in the study. [file 13014_2022_2153_MOESM1_ESM.docx]

| **Antigen** | **Clone** | **Species** | **Dilution** | **Antigen retrieval** | **Company** |
| --- | --- | --- | --- | --- | --- |
| CD3 | F7.2.38 | Rabbit | Prediluted | pH9, 20 min. | Dako IR503 |
| CD4 | 4B12 | Mouse | Prediluted | pH9, 20 min. | Dako IR649 |
| CD8 | C8/144B | Mouse | Prediluted | pH6.1, 30 min. | Dako IR623 |
| PD-L1 | SP263 | Rabbit | Prediluted | pH9, 20 min. | Ventana  790-4905 |
| TIGIT | TG1 | Mouse | 1:100 | pH6.1, 30 min. | ONKO Dianova DIA-TG1 |
| TIM-3 | D5D5R | Rabbit | 1:100 | pH6.1, 30 min. | Cell Signaling 45208 |
| LAG-3 | 17B4 | Mouse | 1:200 | pH6.1, 30 min. | Novus Bio NBP1-97657 |
| Osteopontin |  | Mouse | 1:300 | pH6.1, 30 min. | DSHB  MPIIB10(1) |
| CD96 | NBP2-49491 | Rabbit | 1:500 | pH6.1, 30 min. | Novus biologicals |

Supplementary Table 1: Details about the antibodies used in the study.
